# Supplementary material for: Unraveling Adaptation in Eukaryotic Pathways: Lessons from Protocells
Source: PLoS Comput Biol. 2013 Oct 24;9(10):e1003300. doi: 10.1371/journal.pcbi.1003300 (PMC3812047; doi:10.1371/journal.pcbi.1003300)
Supplement: Text S1 — Supporting information including analytical derivations, additional figures for the two minimal models, fold-change detection, and gradient sensing for the two-component model. (PDF) [file pcbi.1003300.s001.pdf]

Text S1
Supporting Information for:
Unraveling adaptation in eukaryotic pathways: lessons from protocells

Giovanna De Palo[1,2] and Robert G. Endres[1,2]

[1]Department of Life Sciences, Imperial College, London, United Kingdom
[2]Centre for Integrative Systems Biology and Bioinformatics, Imperial College, London, United Kingdom

Contents

1 Single-component model **1**
- 1.1 Adaptation time: simulations 2
- 1.2 Adaptation time: analytical study 4
- 1.3 Fold-change detection 11

2 Two-component model **11**
- 2.1 Adaptation time: simulations and analytical study 13
- 2.2 Fold-change detection 18
- 2.3 Gradient sensing 18

In this *Supporting Information* we present analytical derivations of the properties described in the main text, together with additional figures for the two models, for different input concentrations and receptor-complex sizes. Here, the receptor-complex size is defined by the Monod-Wyman-Changeux model [24]. In particular, the behavior of the adaptation time is explained for the one-component model, both for a special and a general case. This is followed by the formal derivation to demonstrate that the models satisfy fold-change detection. Lastly, we address gradient sensing.

1 Single-component model

The main hypothesis of the work is that adaptation can be obtained even in the absence of energy consumption by the cell, i.e. through equilibration processes. The idea of the main text is to build a model for a "protocell" in which the receptors distributed on the cell membrane are activated (change conformation) by the binding of an extracellular ligand and deactivated by the binding on its

intracellular domain of the same type of ligand, which has diffused through the membrane into the cell.

Figs. S1-S4 show additional adaptation time courses of the one-component model with $A = 1/(1 + e^{N\Delta f})$ from Eq. (3), extended by the receptor-complex size N. In particular, the internal concentration, the single-receptor free-energy difference and the receptor-complex activity are presented for different complex sizes (N=1, 4, 10) and different length of the receptors (r=0.5, 0.7, 0.9). The outer concentrations range from 0 to 0.1mM (Fig. S1), from 0 to 0.5mM (Fig. S2), from 0 to 1mM (Fig. S3), and from 0 to 5mM (Fig. S4). Next, we characterize the properties of the one-component model (for the sake of simplicity we chose $N = 1$).

1.1 Adaptation time: simulations

We can define the adaptation time as the time required for the activity to return to half of the displacement from steady-state level. From the time course of the simulations we can estimate the adaptation time from the activity profile, both for the response to a positive and a negative step in external concentration Δc. Fig. 5 shows the dependence of the adaptation time on the concentration step size and Fig. S5 shows the dependence on the free-energy change for different receptor lengths. The free-energy change δf is calculated as the change of the free-energy difference after and before the external concentration step:

$$\delta f = \ln\left(\frac{1 + \frac{\Delta c}{K_1 + c_0}}{1 + \frac{\Delta c}{K_2 + c_0}}\right) \tag{S1}$$

with Δc the difference between the maximal external concentration c_e and the minimal c_0, and K_1 and K_2 the ligand-dissociation constants of the receptor. In particular, for the external domain of the receptor, K_1 corresponds to the dissociation constant for the *off* state and K_2 for the *on* state, while for the internal binding K_1 correspond to the *on* state and K_2 to the *off* state. In Fig. S5 the adaptation time increases with respect to the free-energy change in response to a positive step, while it decreases for a negative step. We are now going to explain the mathematical reasons of this behavior in our model.

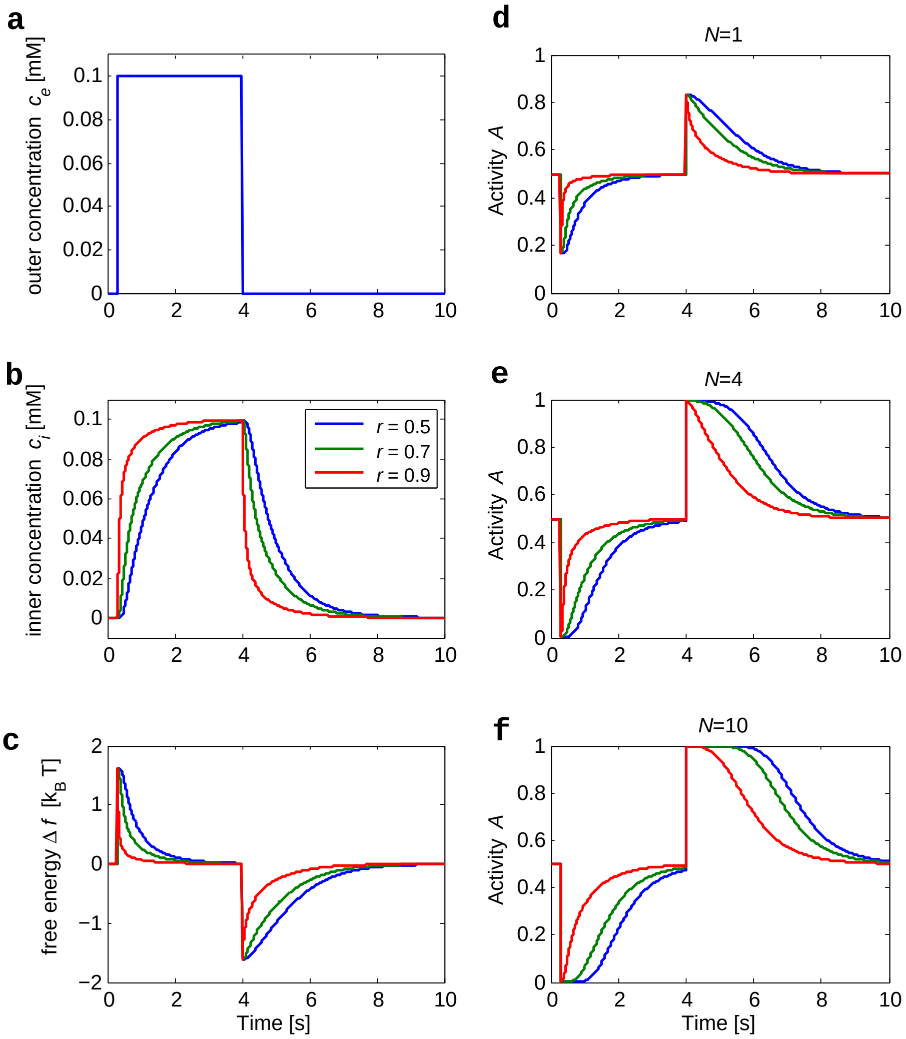

Figure S1: **Adaptation time courses of the one-component model for a 0.1mM step in ligand concentration for background concentration 0mM.** (a) External concentration (input). (b) Internal concentration as a result of diffusion. (c) Free-energy difference of the receptor. (d)-(f) Activity for different receptor-complex sizes N=1, 4, and 10, respectively.

1.2 Adaptation time: analytical study

Using the solution of the diffusion equation, Eq. (1), we define the ratio of concentration differences

$$\frac{\Delta c\,(t)}{\Delta c_0} = \frac{c_i\,(t) - c_e}{c_0 - c_e},$$

and notice that all the concentration time courses obtained for different c_e and c_0 in response to positive and negative steps in external concentration collapse onto a single curve. This allows us to generically analyze the adaptation time (see Fig. S6). As a consequence, the time courses of the internal concentration c_i are symmetrical in response to positive and negative steps in c_e, and are proportional to the maximal external concentration c_e (see Figs. S1-S4b).

Special case. The steady-state of the activity A_{ss} is equal to $1/2$ as can be seen in Figs. S1-S4. For the case in which the activity response saturates we can calculate the adaptation time as the time required for the activity to reach $1/4$ in response to a positive step and $3/4$ in response to a negative step. Based on Eq. (3), the corresponding values for the free-energy difference are $\ln 3$ and $-\ln 3$, respectively. From Eq. (2) we estimate the corresponding internal concentrations

$$c_i\,(A = 1/4) = \frac{(3K_1 - K_2)c_e + 2K_1K_2}{K_1 - 3K_2 - 2c_e} \tag{S2}$$

$$c_i\,(A = 3/4) = \frac{2K_1K_2}{K_2 - 3K_1}. \tag{S3}$$

Note for the first equation we set $c_0 = 0$, and for the second $c_e = 0$ (see Fig. S7).

Next, we would like to see the locations of these two points on the collapsed curve $\Delta c\,(t)\,/\Delta c_0$, in order to compare them and to find out whether their position depends on particular parameters:

$$\frac{\Delta c(A{=}1/4)}{\Delta c_0} = 1 - \frac{(K_2 - 3K_1)c_e - 2K_1K_2}{2c_e^2 + (3K_2 - K_1)c_e} \tag{S4}$$

$$\frac{\Delta c(A{=}3/4)}{\Delta c_0} = \frac{2K_1K_2}{(K_2 - 3K_1)c_e} \tag{S5}$$

We notice that the value of the first point, $\Delta c\,(A = 1/4)\,/\Delta c_0$, increases with increasing external concentration c_e. This means that under our assumptions the adaptation time of the response of the system to increasing positive steps in external concentration decreases (as seen in Fig. 5a). When, instead, we consider the response to increasing negative steps we notice that $\Delta c\,(A = 3/4)\,/\Delta c_0$ decreases with increasing c_e, so the adaptation time consequently increases.

General case. We now take a more general perspective by considering a generic c_0, as well as adding an external contribution Δf_0 to the free-energy difference. The free-energy difference becomes

$$\Delta f = \ln\left(\frac{1 + c_e(t)/K_1}{1 + c_e(t)/K_2}\right) + \ln\left(\frac{1 + c_i(t)/K_2}{1 + c_i(t)/K_1}\right) + \Delta f_0,$$

and the new steady-state value of the activity is given by

$$A_{ss} = \frac{1}{1 + e^{\Delta f_0}},$$

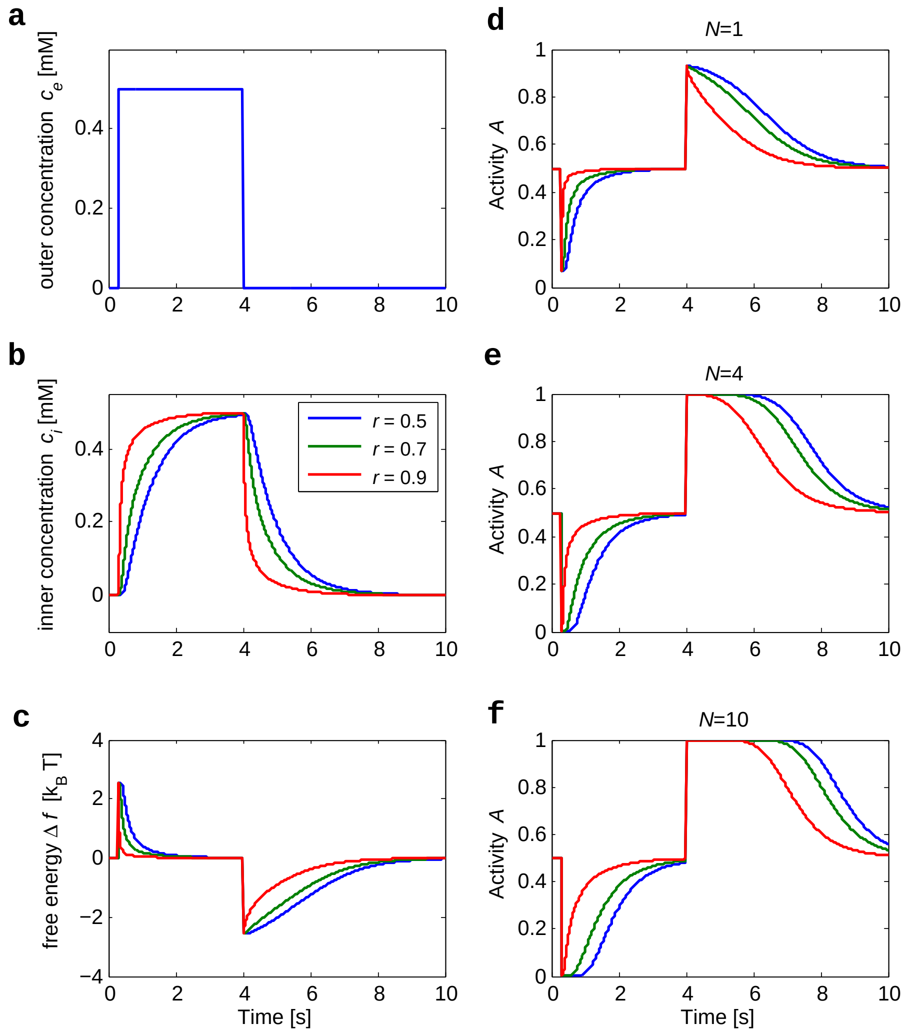

Figure S2: **Adaptation time courses of the one-component model for a 0.5mM step in ligand concentration for background concentration 0mM.** (a) External concentration (input). (b) Internal concentration as a result of diffusion. (c) Free-energy difference of the receptor. (d)-(f) Activity for different receptor-complex sizes N=1, 4, and 10, respectively.

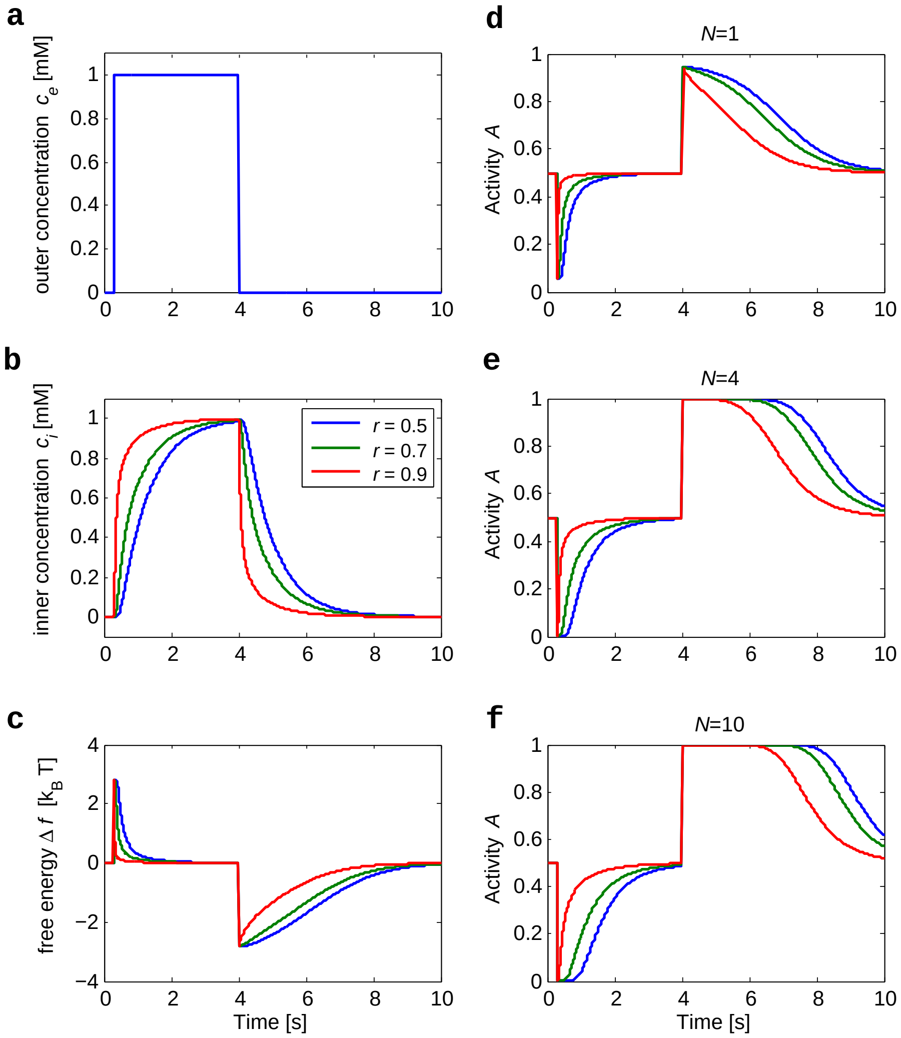

Figure S3: **Adaptation time courses of the one-component model for a 1mM step in ligand concentration for background concentration 0mM.** (a) External concentration (input). (b) Internal concentration as a result of diffusion. (c) Free-energy difference of the receptor. (d)-(f) Activity for different receptor-complex sizes N=1, 4, and 10, respectively.

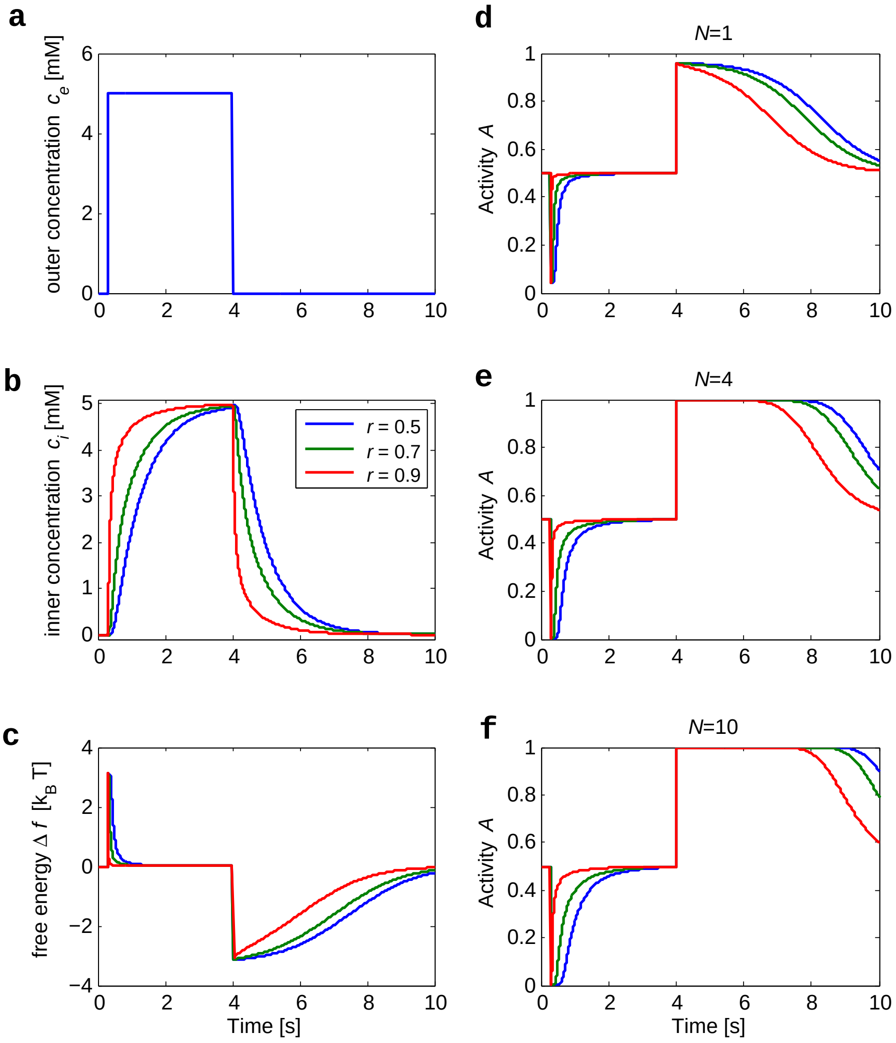

Figure S4: **Adaptation time courses of the one-component model for a 5mM step in ligand concentration for background concentration 0mM.** (a) External concentration (input). (b) Internal concentration as a result of diffusion. (c) Free-energy difference of the receptor. (d)-(f) Activity for different receptor-complex sizes N=1, 4, and 10, respectively.

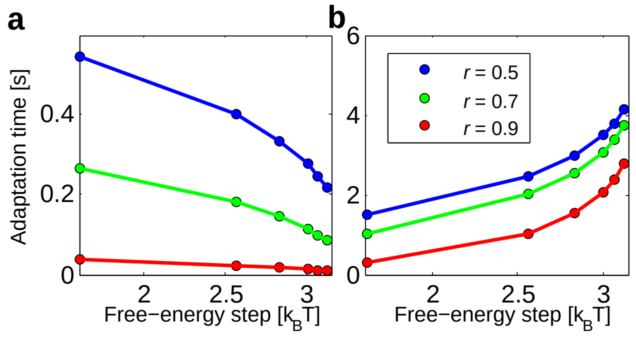

Figure S5: **Dependence of the adaptation time of the one-component model on free-energy step.** (a) Adaptation time as a function of the free-energy difference step, in response to a positive concentration step. (b) Same in response to a negative concentration step. Receptor-complex size $N = 1$.

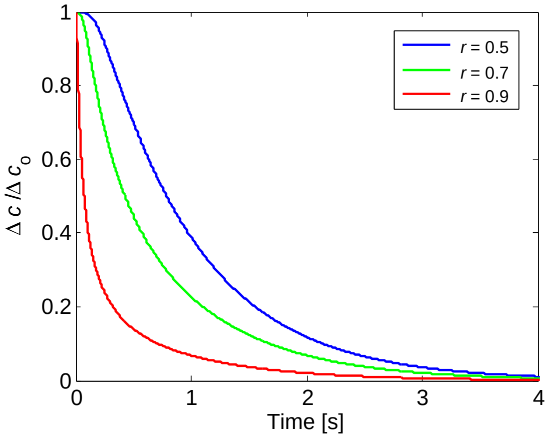

Figure S6: **Collapse of concentration time courses.** $\Delta c/\Delta c_0$ as a function of time for different receptor length r. All the curves resulting from the response to different concentration-step changes collapse onto a single curve (for fixed r). To compare the responses to positive and negative steps in external concentration, we can substitute c_e with c_0 and c_0 with c_e.

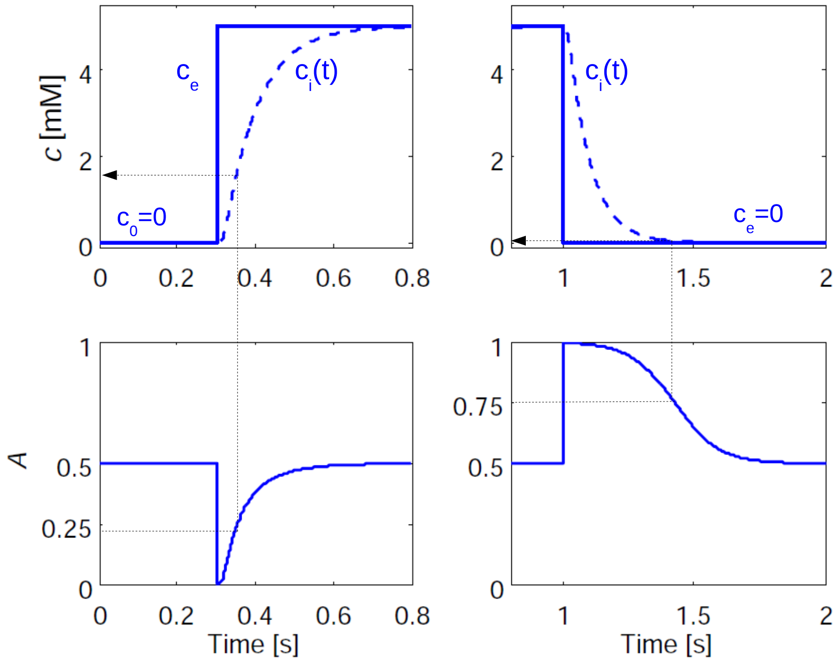

Figure S7: **Procedure for the estimation of the adaptation time of the one-component model.** Assuming that the minimum and maximum of the activity profile A are equal to 0 and 1, respectively, we can define the adaptation time as the difference between the initial time of the change of the stimulus and the time corresponding to $A = 1/4$ (in response to a step-up, left column), and to $A = 3/4$ (for the step-down, right column). This can be done by finding the corresponding amount of the internal concentration c_i and by inserting it in the collapsed curve of Fig. S6, as described in the text.

which is now asymmetric, i.e. $A_{ss} \gtrless 1/2$ for $\Delta f_0 \lessgtr 0$. As before, assuming a saturating response, we can calculate the activity values A_1 and A_2 corresponding to the half-displacement from the adapted steady-state level in response to a positive and negative step in external concentration. The corresponding free-energy values are given by:

$$\Delta f(A = A_1) = \ln(1 + 2e^{\Delta f_0}) \tag{S6}$$

$$\Delta f(A = A_2) = \ln\left(\frac{e^{\Delta f_0}}{2 + e^{\Delta f_0}}\right) \tag{S7}$$

with internal concentrations

$$c_i(A = A_1) = \frac{(K_1 + 2K_1 e^{\Delta f_0} - K_2 e^{\Delta f_0})c_e + K_1 K_2(1 + e^{\Delta f_0})}{K_1 e^{\Delta f_0} - K_2 - 2K_2 e^{\Delta f_0} - (1 + e^{\Delta f_0})c_e} \tag{S8}$$

$$c_i(A = A_2) = \frac{(2K_2 - K_1 + K_2 e^{\Delta f_0})c_0 + K_1 K_2(1 + e^{\Delta f_0})}{K_2 - 2K_1 - K_1 e^{\Delta f_0} - c_0(1 + e^{\Delta f_0})}, \tag{S9}$$

where for the second equation c_e has been substituted with c_0. Calculating the concentration-difference ratios on the collapsed curve

$$\frac{\Delta c(A = A_1)}{\Delta c_0} = \frac{1}{c_e - c_0} \cdot \frac{c_e^2 + (K_1 + K_2)c_e + K_1 K_2}{\frac{K_2 + (2K_2 - K_1)e^{\Delta f_0}}{1 + e^{\Delta f_0}} + c_e} \tag{S10}$$

$$\frac{\Delta c(A = A_2)}{\Delta c_0} = \frac{1}{c_e - c_0} \cdot \frac{c_0^2 + (K_1 + K_2)c_0 + K_1 K_2}{\frac{K_2 - 2K_1 - K_1 e^{\Delta f_0}}{1 + e^{\Delta f_0}} - c_0}, \tag{S11}$$

it is possible to proceed with further considerations. In particular, for Eq. (S10):

- if $c_e \gg c_0$, increasing Δf_0 causes $\Delta c\,(A = A_1)\,/\Delta c_0$ to decrease, thus leading to longer adaptation times. The dependence of the system on the external concentration is not affected (still decreases with increasing c_e, as for the special case);
- if $c_e \simeq c_0$ the assumption of a saturating response does not hold, so we cannot study this case with the above procedure.

For Eq. (S11):

- if $c_e \gg c_0$, $\Delta c\,(A = A_2)\,/\Delta c_0$ increases with increasing Δf_0, which means that the adaptation time decreases. As for the special case, $\Delta c\,(A = A_2)\,/\Delta c_0$ decreases with increasing c_e, leading to longer adaptation times with increasing external concentrations.
- if $c_e \simeq c_0$ as before the saturation assumption is not satisfied.

In summary, in response to a positive step in external concentration, the adaptation time in this minimal model slightly decreases with increasing concentration change, and increases with increasing Δf_0. On the contrary, in response to a negative step in external concentration, the adaptation time increases with increasing c_e and decreases with increasing Δf_0.

1.3 Fold-change detection

An important property most sensory systems fulfill is fold-change detection (FCD), as introduced in the main text. In FCD, the response depends only on the ratio of the initial and final values of the input concentration step, and not on their absolute values (i.e. scaling the input profile by a multiplicative factor does not affect the response of the model). Considering our minimal model as described in Eq. (S1), the change in the free-energy difference only depends on the ratio $\left(1 + \frac{\Delta c}{K_1 + c_0}\right) / \left(1 + \frac{\Delta c}{K_2 + c_0}\right)$. This means when choosing $K_1 \ll c_0 \ll K_2$, the response profile only depends on the ratio $(c_e - c_0)/c_0$ and consequently FCD holds. The corresponding simulations for demonstrating this behavior are shown in Fig. 5.

2 Two-component model

As explained in the main text, the one-component model can be extended by including a second transmembrane protein to approach more closely real biological mechanisms without loosing much of its tractability. In this two-component model, the stimulus activates the receptor, thus causing the unbinding of two smaller cytoplasmic subunits that we call a and b. The subunit a is smaller than b and hence diffuses faster. A second transmembrane protein possesses two binding sites for the a and b subunits and is responsible for the final signaling. This second protein decreases its activity upon the binding of a, and increases it with the subsequent binding of b. We assume that the effects of a and b binding to the second signaling protein exactly compensate each other, and that a and b have the same stoichiometry, both for binding the receptor and the signaling protein. Moreover, we hypothesize that the number of activated receptors is much higher than the number of signaling proteins, thus the concentrations of a and b depend only on the activity of the receptor and are unaffected by binding and unbinding to the signaling protein.

Figs. S8-S11 show results of the simulations obtained in response to different steps in the outer concentration, for different complex sizes (N_2=1, 4, 10) and lengths for the second signaling protein (r=0.5, 0.7, 0.9). Specifically, outer concentrations range from 0 to 0.1mM (Fig. S8), from 0 to 0.5mM (Fig. S9), from 0 to 1mM (Fig. S10), and from 0 to 5mM (Fig. S11). Generally, panel a shows the concentrations of a and b secreted by the first receptor, and panel b shows the ratio in concentrations of a and b perceived by the signaling protein after diffusion. In panel c, the free-energy difference Δf_2 for the signaling protein is presented. On the right, final signaling activities profiles for the second transmembrane protein and different complex sizes are shown.

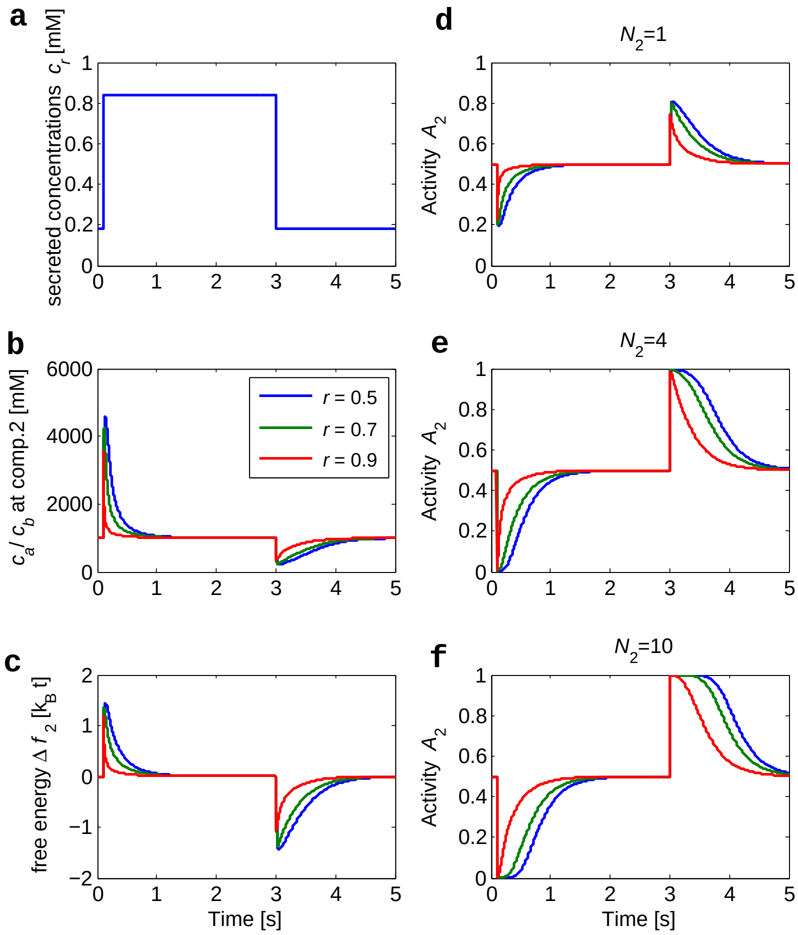

Figure S8: **Adaptation time courses of the two-component model for a 0.1mM step in ligand concentration for background concentration 0mM.** (a) Secreted concentration of a and b. (b) Ratio c_a/c_b bound to the second component, after diffusion. (c) Free-energy difference of the signaling protein. (d)-(f) Activity for different receptor-complex sizes of the signaling protein N_2=1, 4, 10, respectively.

2.1 Adaptation time: simulations and analytical study

As done for the one-component model, it is possible to study the adaptation time and its dependence on the amplitude of the concentration step. Specifically, Figs. S8-S11 show the activity of the signaling protein $A_2 = 1/(1 + e^{N_2 \Delta f_2})$ in response to stimulus $c_e(t)$, with N_2 the size of the signaling complex (cf. Eq. (7)). Concentration c_r is the released concentration of a (and hence b) at the receptor. For simplicity, we assume this released concentration to be constant during the stimulation, i.e. unaffected by diffusion. The results are summarized in Fig. S12, in which the adaptation time is shown as functions of the concentration step in $c_r(t)$ and the step in the free-energy difference, given by

$$\delta f_2 = \ln\left(\frac{1+\frac{c_{r,\max}}{L_D}}{1+\frac{c_{r,\min}}{L_D}}\right), \tag{S12}$$

where $c_{r,\min}$ and $c_{r,\max}$ represent, respectively, the released concentrations of a and b before and after the step in the activity of the receptor (Figs. S8-S11a).

Notice from Fig. S12 that the adaptation time is almost independent of the change in the external concentration in response to a positive step, while it increases with increasing concentration for a negative step. Also for this model, it is possible to obtain analytical understanding for this dependence. From Eq. (7) we can calculate the free-energy differences corresponding to $A_2 = 1/4$ and $A_2 = 3/4$ to be $\Delta f_2 = \ln 3$ and $\Delta f_2 = -\ln 3$, respectively. Assuming that diffusion of a is fast enough, relative to b, to be approximated with $c_r(t)$, we can calculate from Eq. (6)

$$c_b(A = 1/4) = \frac{c_{r,\max} - 2L_D}{3} \tag{S13}$$

$$c_b(A = 3/4) = 2L_D + 3c_{r,\min}. \tag{S14}$$

Also in this case, we can consider the collapsed curves

$$\frac{\Delta c_a}{\Delta c_r} = \frac{c_a(t) - c_r(t)}{c_{r,0} - c_r(t)} \tag{S15}$$

$$\frac{\Delta c_b}{\Delta c_r} = \frac{c_b(t) - c_r(t)}{c_{r,0} - c_r(t)}, \tag{S16}$$

where $c_r(t)$ represents the current value of the released concentration and $c_{r,0}$ the value before the step (thus corresponding to $c_{r,\min}$ in the case of a positive step and to $c_{r,\max}$ for a negative step). These curves are qualitatively similar to the curve presented in Fig. S6, in which all the different concentrations for the positive and negative steps superimpose. When calculating the values corresponding to $A = 1/4$ and $A = 3/4$, under the approximation of $a \simeq c_r$, Eq. (S15) becomes negligible, and we can thus consider only Eq. (S16). Assuming that $c_{r,\max} \gg c_{r,\min}$ and $c_{r,\max} \gg L_D$, we have

$$\frac{\Delta c(A = 1/4)}{\Delta c_r} = \frac{c_b(A = 1/4) - c_{r,\max}}{c_{r,\min} - c_{r,\max}} = \frac{2}{3} \tag{S17}$$

$$\frac{\Delta c(A = 3/4)}{\Delta c_r} = \frac{c_b(A = 3/4) - c_{r,\min}}{c_{r,\max} - c_{r,\min}} = \frac{2(L_D + c_{r,\min})}{c_{r,\max}}. \tag{S18}$$

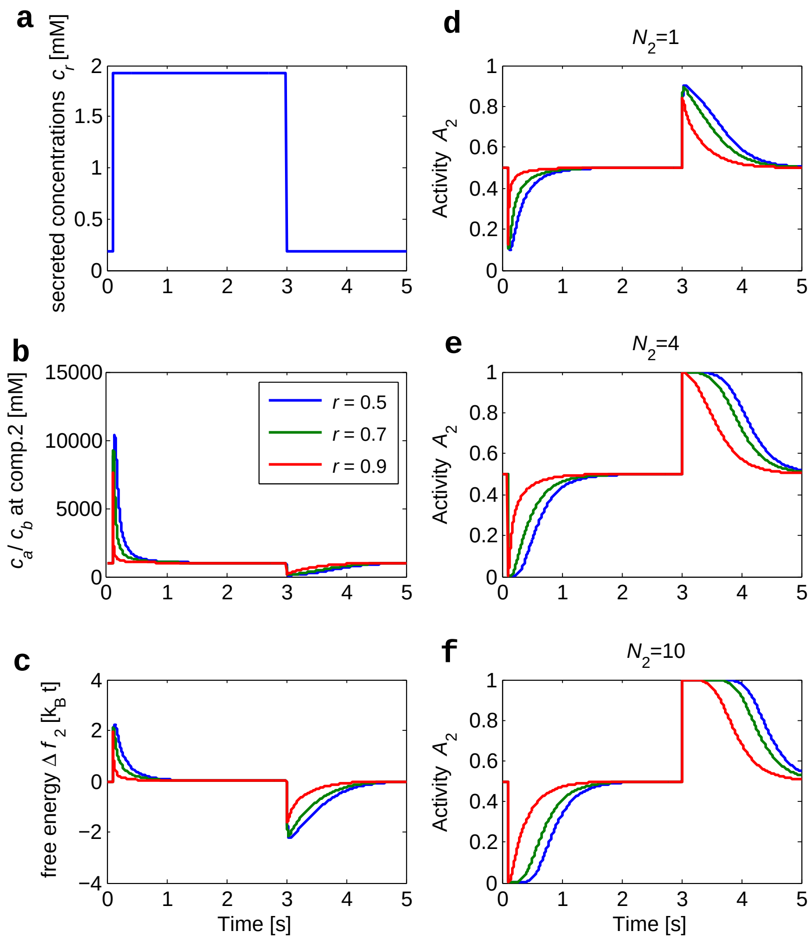

Figure S9: **Adaptation time courses of the two-component model for a 0.5mM step in ligand concentration for background concentration 0mM.** (a) Secreted concentration of a and b. (b) Ratio c_a/c_b bound to the second component, after diffusion. (c) Free-energy difference of the signaling protein. (d)-(f) Activity for different receptor-complex sizes of the signaling protein N_2=1, 4, 10, respectively.

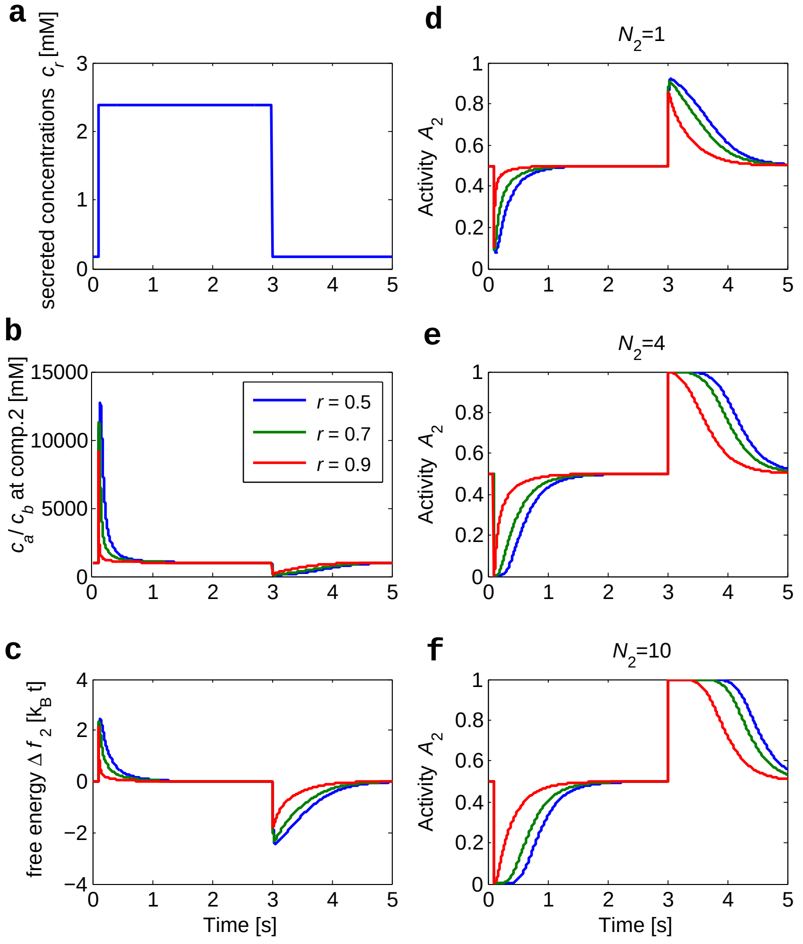

Figure S10: **Adaptation time courses of the two-component model for a 1mM step in ligand concentration for background concentration 0mM.** (a) Secreted concentration of a and b. (b) Ratio c_a/c_b bound to the second component, after diffusion. (c) Free-energy difference of the signaling protein. (d)-(f) Activity for different receptor-complex sizes of the signaling protein N_2=1, 4, 10, respectively.

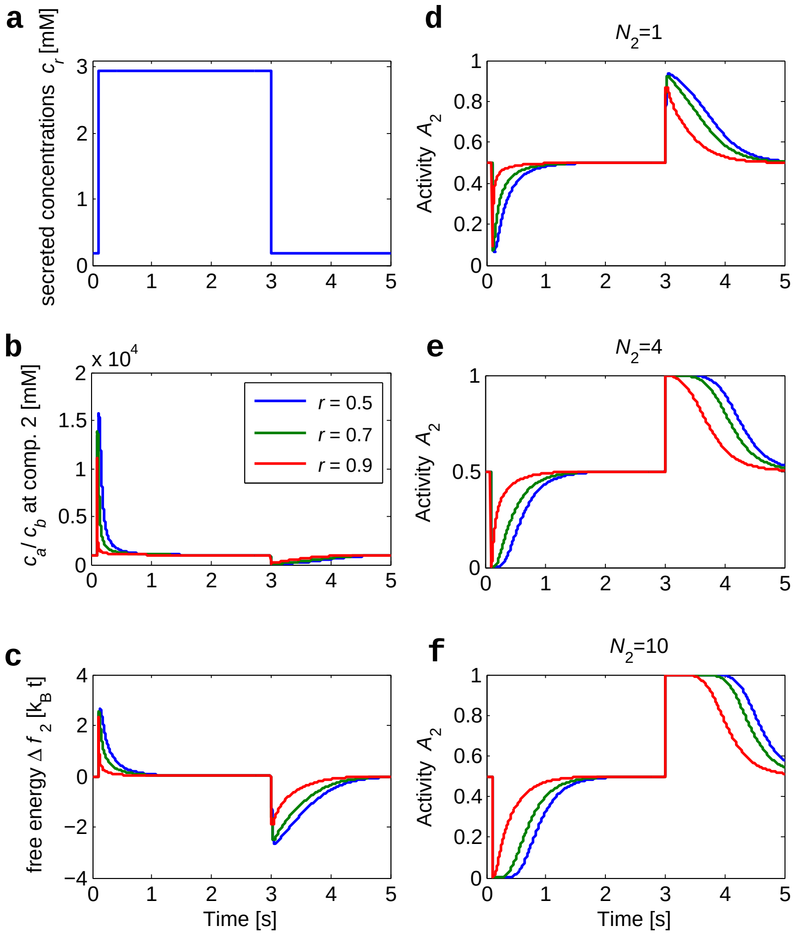

Figure S11: **Adaptation time courses of the two-component model for a 5mM step in ligand concentration for background concentration 0mM.** (a) Secreted concentration of a and b. (b) Ratio c_a/c_b bound to the second component, after diffusion. (c) Free-energy difference of the signaling protein. (d)-(f) Activity for different receptor-complex sizes of the signaling protein N_2=1, 4, 10, respectively.

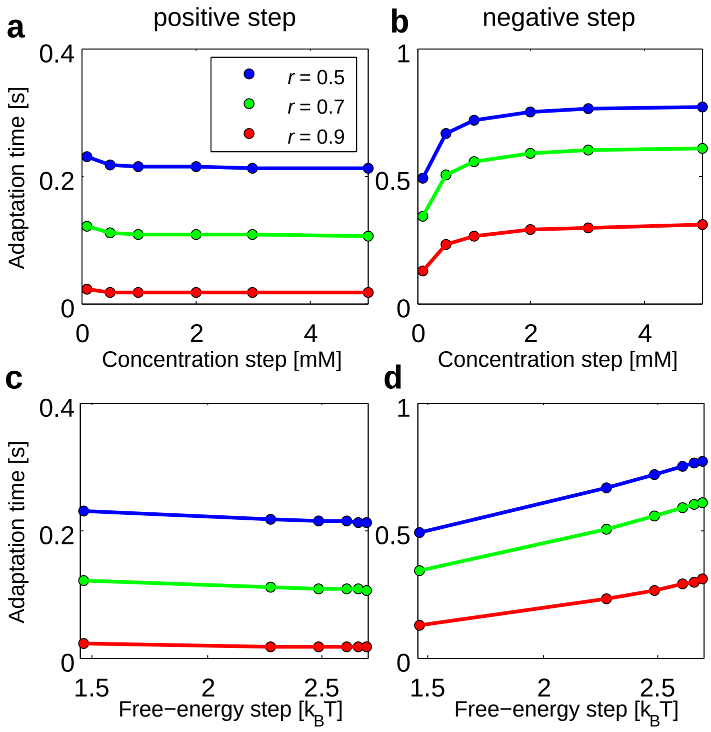

Figure S12: **Dependence of the adaptation time of the two-component model on external concentration and free-energy step.** Response to steps in the external concentrations c_e, changing from 0 to 0.1, 0.5, 1, 2, 3, 5mM and back to 0mM. Complex size N_2=1. (left panels) Adaptation time in response to a positive step in external concentration c_e as a function of concentration c_e (a) and change in free-energy difference δf_2 (c). (right panels) Adaptation time in response to a negative step in external concentration c_e as a function of concentration c_e (b) and change in free-energy difference δf_2 (d).

Regarding the positive step response, the point corresponding to $A = 1/4$ on the collapsed curve does not depend on c_r (under our assumptions), and consequently on the external concentration c_e. This explains the (nearly) constant adaptation time in response to different positive steps of external concentration. Note instead that $c_{r,\min}$ corresponds to $c_e = 0$ independent of the amplitude of the step. Thus the value of the point described by Eq. (S18) for the negative step decreases with increasing $c_{r,\max}$ (and thus with increasing maximal external concentration c_e), and consequently the adaptation time increases. These results can be observed in Fig. S12.

2.2 Fold-change detection

Similar to the one-component model, the two-component model also satisfies FCD. Indeed, we can consider the change in free-energy difference δf_2 of Eq. (S12) and, assuming the diffusion of a much faster than that of b, approximate c_a with the maximal value of the released concentration and c_b with the minimal. The released concentration of a and b corresponds to the activity of the receptor A_1 rescaled by their proportionality factor (10^{-2}). The equation we obtain from this substitution is

$$\delta f_2 = \ln\left(\frac{1+\frac{\Delta c}{c_0+P}}{1+\frac{\Delta c}{c_0+Q}}\right),$$

in which, for special case $L_D = K_{\text{on}}$,

$$P = \frac{K_{\text{on}}K_{\text{off}} + 100K_{\text{on}}^2K_{\text{off}} + 100K_{\text{on}}^2K_{\text{off}}e^{\sigma}}{100K_{\text{on}}K_{\text{off}} + 100K_{\text{on}}^2e^{\sigma} + K_{\text{off}}},$$

$$Q = \frac{K_{\text{on}}K_{\text{off}} + K_{\text{on}}K_{\text{off}}e^{\sigma}}{K_{\text{off}} + K_{\text{on}}e^{\sigma}}.$$

As for the one-component model, we can find values for c_0 with $P \ll c_0 \ll Q$, such that the conditions for FCD are satisfied (see Fig. S13 for an example).

2.3 Gradient sensing

As described in the main text for the one-component model, we performed simulations for the case of a uniform spatial gradient in ligand concentration also for the two-component model. The results are presented in Fig. S14. Also this model shows perfect adaptation in the activity time courses, and the directional sensing exhibits a small delay as compared to the one-component model because the activation is due to the diffusion of the a subunit, which is fast but not instantaneous as in the one-component model.

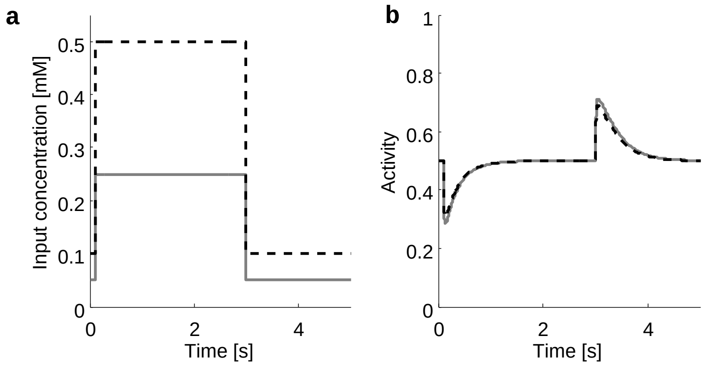

Figure S13: **Fold-change detection in the two-component model.** (a) Input concentration and (b) activity time courses for $c_0 = 0.05$mM and $c_e = 5 \cdot c_0$ (gray lines), and $c_0 = 0.1$mM and $c_e = 5 \cdot c_0$ (black dashed lines).

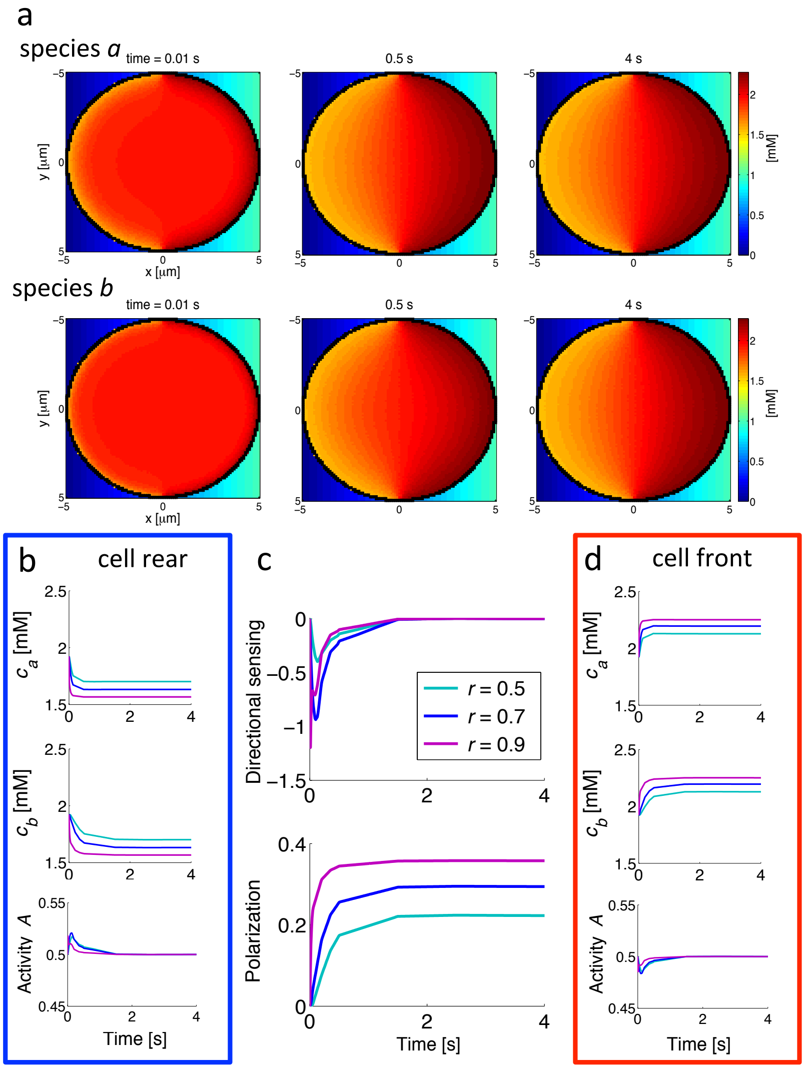

Figure S14: **Spatial gradient sensing in the two-component model.** (a) Extracellular ligand concentration and intracellular a (top) and b (bottom) concentrations in the x-y plane at different times. (b) Internal a and b concentrations (top) and activity (bottom) time courses for different receptor lengths at the cell rear at location (-5 $\mu m + l_{rec}$, 0). (c) Directional sensing (top) and polarization in the concentration of b (bottom) for different receptor lengths. (d) Internal a and b concentrations (top) and activity time courses (bottom) for different receptor lengths at the cell front at location (5 $\mu m - l_{rec}$, 0).
